# RNAseq, transcriptome analysis and identification of DEGs involved in development and ripening of *Fragaria chiloensis* fruit

**DOI:** 10.3389/fpls.2022.976901

**Published:** 2022-09-20

**Authors:** Carlos Gaete-Eastman, Yazmina Stappung, Sebastián Molinett, Daniela Urbina, María Alejandra Moya-Leon, Raúl Herrera

**Affiliations:** Laboratorio de Fisiología Vegetal y Genética Molecular, Instituto de Ciencias Biológicas, Universidad de Talca, Talca, Chile

**Keywords:** Illumina sequencing, Chilean strawberry fruit, fruit ripening, cell wall enzymes, ABA genes, flavonoids genes

## Abstract

*Fragaria chiloensis* (Chilean strawberry) is a native species that produces fruit with an exotic pinkish color and a fruity aroma. It has a non-climacteric pattern of fruit ripening, and it is the mother of the commercial *Fragaria* x *ananassa*. The ripening of *F. chiloensis* fruit seems stimulated by ABA, and a complete set of genes participate in its softening, color, and aroma development. In addition, a set of transcription factors regulate the entire process, but few of them have been described. Over the last two decades, RNA-seq was used to identify genes at three fruit development/ripening stages, named C2 (unripe, large green) to C4 (full ripe), in whole fruit and fruit without achenes. A total of 204,754 contigs were assembled considering all samples, obtaining an N50 of 1.125 bp. Differentially expressed genes (DEGs) between two samples were identified, obtaining a total of 77,181 DEGs. Transcripts for genes involved in ABA biosynthesis present high and differential expression during the C2, C3, and C4 stages. Besides, contigs corresponding to ABA receptors, which interact with a regulatory network, are also differentially expressed. Genes associated with cell wall remodeling and those involved in flavonoid synthesis were also differentially expressed. An interaction network was built considering differentially expressed genes for the phenylpropanoid and flavonoid molecular pathways and having FcMYB1 as a transcription factor regulator. Identifying key genes could give an option to control the ripening of this non-climacteric fruit.

## Introduction

*Fragaria chiloensis* (L.), a mill, is a non-climacteric fruit with many valuable traits, such as a fruity aroma, a pinkish color, and high nutritional content (Letelier et al., 2020). The species has been established in different edaphoclimatic conditions in Chile. However, the major production is around the valleys close to Nahuelbuta mountain (cities of Purén and Contulmo) (Letelier et al., 2020). As a *Fragaria* species, the Chilean strawberry fruit is unique because the edible flesh is the enlarged receptacle tissue. The true fruit is the numerous dry achenes on the receptacle surface. The exchange of molecules between achenes and receptacles can coordinate the development and ripening of the fruit. In this sense, phytohormones produced by achenes are essential for the ripening of fruit receptacles, but little is known about the influence of achenes signals on fruit ripening in Chilean strawberries.

The suppression subtractive hybridization (SSH) strategy was used in our first attempt to unravel genes involved during the development and ripening of *Fragaria chiloensis* fruit (Pimentel et al., 2010). A set of 1,807 genes were isolated from six SSH libraries expressed during ripening, and 13 of them were analyzed by qPCR to validate their role in fruit development and ripening. Three genes, *FcPL, FcPG*, and *FcEG*, showed a high accumulation of transcripts at the late fruit ripening stages and are specifically expressed in fruits (Pimentel et al., 2010). Interestingly, auxin-related genes and the transcription factor MADS1 were also differentially expressed at the late fruit ripening stages but showed transcript accumulation in other tissues like flowers and runners.

Nowadays, transcriptome characterization through RNA-seq can achieve the expression profile of a larger number of genes, giving a more detailed insight into the molecular mechanism involved. Sequences can be efficient *de novo* assembled in organisms with reference genomes. A major interest has concentrated on studying the commercial *F. x ananassa* and the diploid *Fragaria vesca* (Hollender et al., 2014; Chen et al., 2016; Wang et al., 2017). These broad transcriptomic studies allowed the identification of differentially expressed genes during the development and ripening of fruit. The roles of plant hormones have been studied by applying the plant hormones abscisic acid (ABA) or auxin to the fruit (Li et al., 2015; Chen et al., 2016). Interestingly, different genes and metabolic pathways were identified when ABA and auxin were used during the fruit ripening of strawberries. The key role of ABA and auxins has been shown in the ripening of strawberry fruits, even though different experimental approaches have been used (Li et al., 2015; Chen et al., 2016).

*Fragaria chiloensis* (L.) Mill, or the Chilean strawberry, is a native fruit of Chile (Letelier et al., 2020). It is appreciated for its good organoleptic qualities, being its sweet and pleasant aroma, the main characteristic of this non-climacteric fruit, in addition to its big fruit size, resistance to pathogens, and better sustainability to soil salinity and low temperature (González et al., 2009, 2013). Furthermore, *F. chiloensis* is the maternal relative and, therefore, the gene source of the commercial strawberry (*Fragaria* × *ananassa*). *F. chiloensis* has the potential to be developed as a new exotic berry in the world market (Retamales et al., 2005). However, the fruit is highly perishable as its rapid softening alters the texture and negatively influences its postharvest life and quality (Perkins-Veazie, 1995; Figueroa et al., 2008).

In this study, RNAseq analysis was performed in *F. chiloensis* considering three different fruit stages and two different types of samples: whole fruit (receptacle with achenes) and only receptacles (without achenes). To understand the molecular mechanisms underlying the differential contribution of achenes and receptacles during the ripening of this strawberry fruit, we performed a comprehensive transcriptome analysis at three developing/ripening fruit stages: immature-large green (C2), turning (C3), and ripe (C4) stages. These results allow the understanding of the molecular regulation of fruit ripening in this non-climacteric species and provide pieces of evidence of the participation of achenes and receptacle tissues in ripening.

## Materials and methods

### Fruit samples

*Fragaria chiloensis* fruit samples were collected from commercial orchards in Purén (the Araucania region, Chile) and immediately transported to the laboratory. Fruit samples were segregated according to external phenotype into three developing and ripening fruit stages as described by Figueroa et al. (2008): C2, large green (large size fruit with red achenes); C3, turning (large size fruit with white receptacle and red achenes) and C4, ripe fruit (full-size fruit with pink receptacle and red/brown achenes). Receptacle fruit samples were obtained after the careful remotion of the achenes from the fruit surface with the help of tweezers. Complete fruit and deachened (receptacle) fruit samples were cut into pieces, frozen under liquid nitrogen, and stored at −80°C as bulks until their use.

### RNA extraction, library construction, and Illumina sequencing

Total RNA was extracted from representative bulks of complete fruit stages (C2, C3, C4) and receptacle samples (RC2, RC3, RC4) using the methodology described by Pimentel et al. (2010). RNA samples were sent to the Next Generation Sequencing (NGS) division at Macrogen Inc. (Seoul, Republic of Korea). One RNA sample per condition was subjected to the library construction protocol at Macrogen and then sequenced with the Illumina HiSeq 2000 platform; 100-bp paired-end reads were generated.

### Sequence data analysis and assembly

Raw sequence data from high-throughput NGS sequencing services were subjected to quality control using the software FastQC (http://www.bioinformatics.babraham.ac.uk/projects/fastqc/). The quality of each sample was checked based on the analysis of per base sequence quality (value >20), per sequence quality score (value of 38), per sequence G.C. content (normal distribution/46%), and percentage of over-represented sequences. Three sequences were identified over the threshold and identified as metallothionein-like proteins.

RNA-seq *de novo* assembly was performed at the NGS division of Macrogen Inc. by using Trinity (http://trinityrnaseq.sourceforge.net). The sequences for this project were loaded into BioProject (ID PRJNA849949).

### Functional annotation

**Figure 1:**
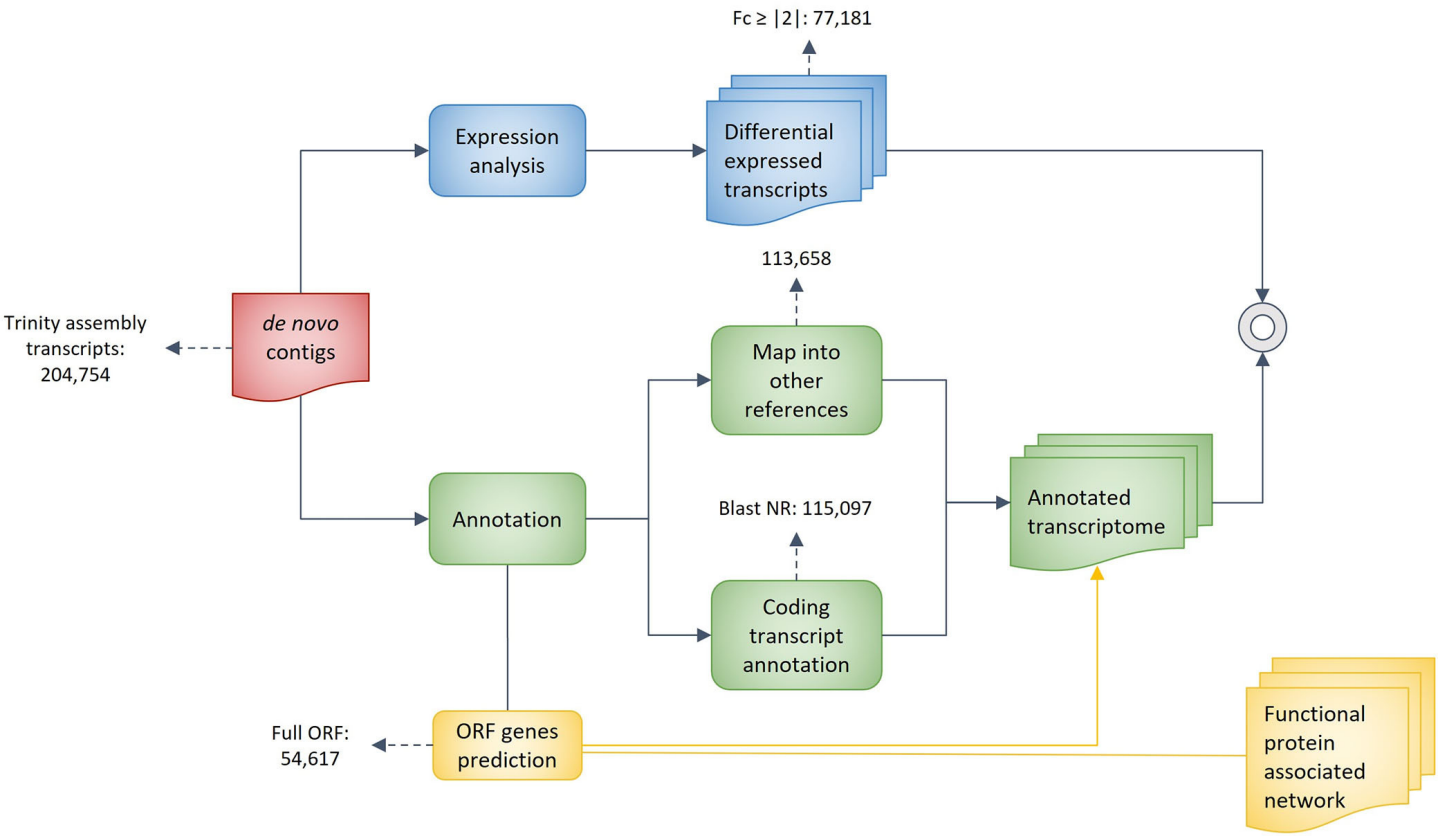
Flow diagram of gene annotation using transcriptomic reference genome. de novo assembled contigs were annotated using four reference genomes and NR database. Full ORF predicted genes from annotation stage were used for functional protein associated network.

Several complementary approaches were employed to annotate the unigenes (Figure 1). The *de novo* obtained sequences were analyzed using the Basic Local Alignment Search Tool (BLAST) (Altschul et al., 1990), and the sequences were compared against reference genomes of four species: three from the Rosaceae family (apple, *Malus domestica 1.0*; peach, *Prunus persica 1.0*; woodland strawberry, *Fragaria vesca 1.1*) and poplar (*Populus trichocarpa 2.0*). Then the genes were functionally annotated under gene ontology terms (G.O., http://www.geneontology.org). Sequences with hits from the output analysis were used to obtain a tentative annotation, which was manually confirmed for a subset of sequences under study in the present work.

According to Trinity methodology, abundance estimation of transcripts was determined by the RSEM alignment-based quantification method, comparing two different sample conditions at a time and obtaining the relative abundance separately for each sample. The normalized expression metrics were reported as FPKM (fragments per Kilobase transcript length per million fragments mapped).

Two validation strategies were used with the assembled data. First, a pair-wise sequence alignment of 7 selected ripening-related genes from *F. chiloensis*, previously published and experimentally tested, was performed against the RNA seq data. Secondly, a sequence mapping of SSH libraries previously reported by Pimentel et al. (2010) against the RNA seq was performed.

### Analysis of expression

The analysis of differentially expressed genes was performed by comparing the following pairs of sequenced libraries: C2 vs. C3; C3 vs. C4; C2 vs. C4; RC2 vs. RC3; RC3 vs. RC4; RC2 vs. RC4; C2 vs. RC2; C3 vs. RC3; C4 vs. RC4. In all these comparisons, the first library (underlined) was the control, and the second was the test library. Fold change and hierarchical clustering were the statistical methods used in the nine comparisons described, and up/down DEGs were counted.

### RT-qPCR analysis

RNA extractions from intact (C samples) and de-achened fruit (R.C. samples) were followed by cDNA (complementary DNA) synthesis, as previously reported (Opazo et al., 2010). In short, 8 g of fruit samples detailed above were extracted using the CTAB method (Chang et al., 1993). Then, total RNA samples were treated with DNase I (Invitrogen) and cleaned up with the RNeasy Plant Mini Kit (Qiagen). Finally, cDNA synthesis was performed using the First Strand cDNA Synthesis kit (Fermentas). RT-qPCR analyses were performed as previously described (Opazo et al., 2013). Briefly, three biological replicates were used for each sample analyzed. All the primers were designed against (or at least one of them), 3'-UTR regions. qPCR reactions were performed using Maxima SYBR Green qRT-PCR Master Mix (Fermentas) following the manufacturer's recommendations in Stratagene Mx300P (Agilent Technologies). Relative expression levels of each gene under study, representing the mean between three biological replicates, were calculated using the method described by Pfaffl (2001) and employing *FcGAPDH1* as a normalizer.

### Heatmap analysis

A color-coded two-dimensional mosaic describing the whole expression matrix (samples vs. gene targets) was built according to Sun and Li (2013). Each tile was colored with different intensity levels according to the preprocessed data expression. Gene expression values can be visualized with the color density ranging from the least expressed (blue) to the most expressed (red).

### String interaction network

String is a database where known and predicted direct (physical) interactions, as well as indirect (functional) interactions, can be established based on co-expression, co-localization, text-mining, and others (Szklarczyk et al., 2017, 2019). The DEGs involved in synthesizing flavonoids and FcMYB were picked, and the web server was interrogated to uncover potential protein-protein association networks. The database was interrogated for the last time in December 2021.

## Results

*Fragaria chiloensis* fruit samples were harvested by local farmers in Purén, in the Araucania region of Chile. Fruits from different development and ripening stages were collected and separated into three different groups.

The NGS sequencing data performed transcriptomic analysis on the three fruit development stages. More than sixty million pair-end reads were generated for each sample (Supplementary Table 1). Because there is no reference genome for *Fragaria chiloensis*, a strict assembly pipeline for annotation and validation was applied (Figure 1). A total of 204,754 tentative contigs were assembled considering all libraries, with an N50 of 1,125 bp (Table 1). Bearing in mind that all libraries were sequenced on one line of Illumina HiSeq2000, the total throughput per library was homogeneous and near the maximum performance of 35 Gb per line. Paired-end reads of 101 bp were obtained for each sample constructed. Finally, read quality, trimming, and the check for no contaminant sequences were examined. The quality of each library was performed by: analysis of per base sequence quality (value >20), per sequence quality score (value of 38), per sequence G.C. content' (normal distribution fitting indicating no contamination with foreign DNA), and percentage of overrepresented sequences (3 sequences over the threshold: metallothionein-like protein). Two strategies were used to annotate all the sequences: first, four reference genomes were used, and second, all sequences were BLAST locally against the non-redundant (nr) database.

**Table 1:** Summary of *de novo* transcriptome assembly.

**Assemble statistics**	**Merge**
The total length of contigs	142,626,488
Total number of contigs	204,754
Max length bp	13,470
Min length bp	201
N90 bp	275
N80 bp	389
N70 bp	565
N60 bp	812
N50 bp	1,125

Information of total contigs and general features were obtained after de novo transcript assembly using Trinity software (2011-11-26 version).

**Figure 2:**
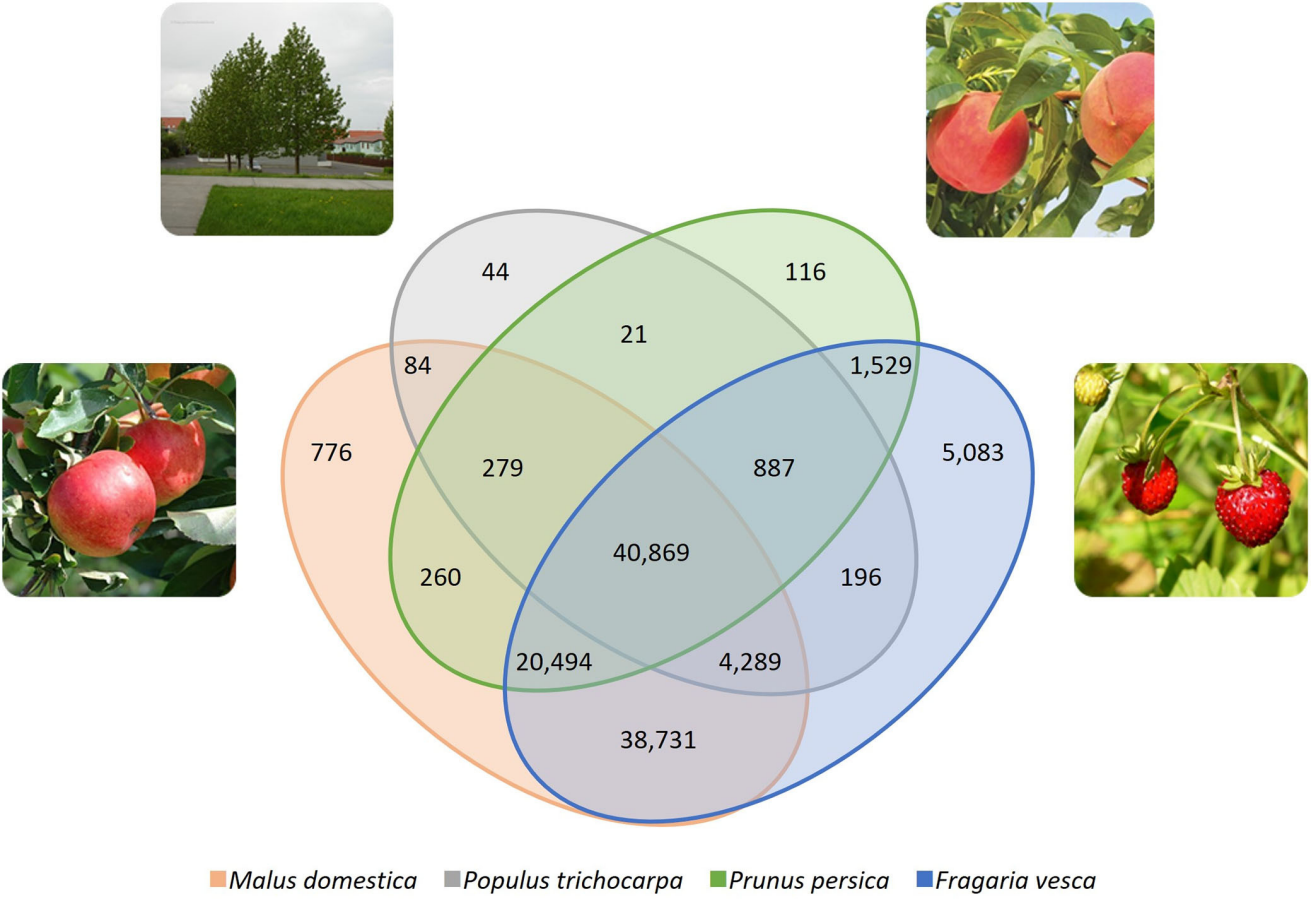
Venn diagram describing the representation of annotated genes within the different reference genomes. Assembled contigs were subjected to BLASTx analysis against three Rosaceae reference genomes (apple, *Malus domestica*; peach, *Prunus persica*; strawberry, *Fragaria vesca*) and that of poplar (*Populus trichocarpa*). A total of 98,710 (48%) *Fragaria chiloensis* transcripts were matched against the reference genomes using a cutoff e value of 1e^−5^. Reference genomes were obtained from Phytozome v. 10 betas (http://phytozome.jgi.doe.gov).

The functional annotation stage was performed through the mapping comparison to three Rosaceae species (apple, peach, and woodland strawberry) and poplar, obtaining a reference of 113,658 contigs (Figure 2). Approximately 20 % of the total assembled contigs were assigned a corresponding cross-entry within the four reference genomes employed. The Fragaria genome contributes the largest of the annotations of the *F. chiloensis* transcriptome, contributing 55% of the total of sequenced contigs. On the other hand, 5,083 annotated contigs were unique between *F. vesca* and *F. chiloensis*.

The validation of assembled data was contrasted with seven selected ripening-related genes from *F. chiloensis*, indicating a complete coverage of the sequence and a very high sequence identity (Supplementary Table 2). Additionally, the alignment of the ESTs from the SSH library (Pimentel et al., 2010) with contigs from the RNA seq transcriptome allows the mapping of complete coverage sequences, in which 67% of ESTs share between 95 and 100% of sequence identity.

**Figure 3:**
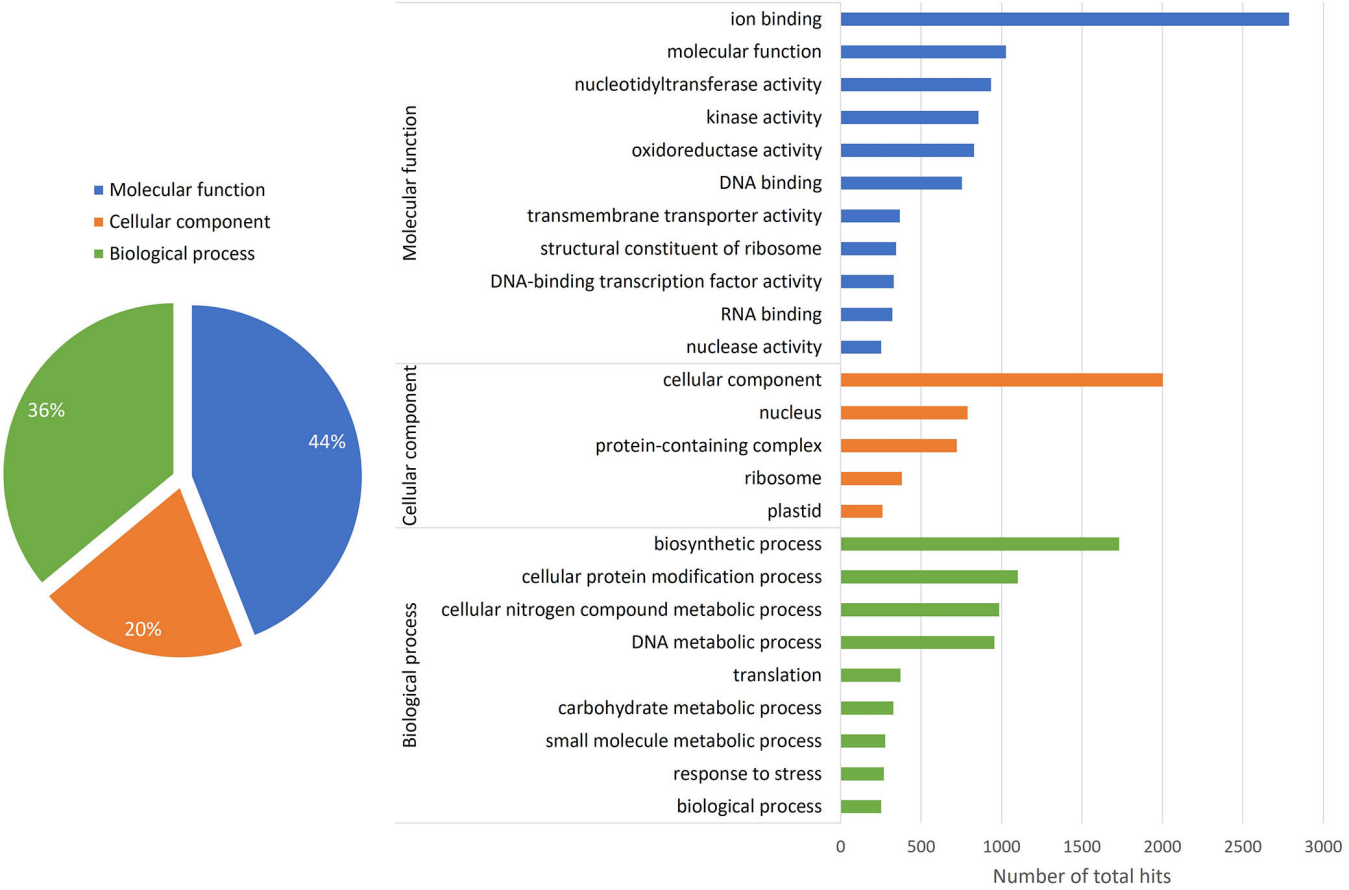
Classification of contigs under Gene Ontology terms. A total of 11,825 contigs were assigned with G.O. terms within the three main categories (Biological process, Cellular components, and Molecular function) and subdivided into their corresponding subcategories. Only relevant G.O. categories (≥ 100 hits per subcategory) are shown.

The annotation through the N.R. database provided 115,097 contigs, which corresponded to 56% of the total sequenced contigs (Figure 1). Also, an enrichment analysis by Gene Ontology (G.O.) assigned at least one G.O. term to 48.6% of the sequences (Figure 3). The G.O. categories were molecular function (44%), biological process (36%), and cellular component (20%). The “ion binding” subcategory is strongly represented in the molecular function category, followed by molecular function. In the cellular component category, some subcategories that were highly represented included cellular components, nuclei, and protein-containing complexes. In the case of the biological process category, the most represented subcategory is a biosynthetic process. Other important subcategories are the cellular protein modification process, cellular nitrogen compound metabolic process, and DNA metabolic process.

### Analysis of differentially expressed genes

To perform an expression analysis, all those assembled contigs having at least one zero FPKM value were excluded from this study, leaving a final total of 77,181 contigs for the analysis (Supplementary Figure 1). The conditions were comparable after performing the distribution of normalized FPKM values before and after log2 transformation, and with or without quantile normalization. Then, hierarchical clustering was performed using the correlations between stages, which grouped more similar samples of this study (Supplementary Figure 2). Interestingly, the C3 stage (sample corresponding to whole fruit at turning stage) grouped with C2 and RC2 stage (samples corresponding to large green whole fruit and receptacle, respectively); while RC3 stage (sample corresponding to receptacle fruit at turning stage) grouped with C4 and RC4 stage (samples corresponding to a ripe whole and receptacle fruit, respectively).

**Figure 4:**
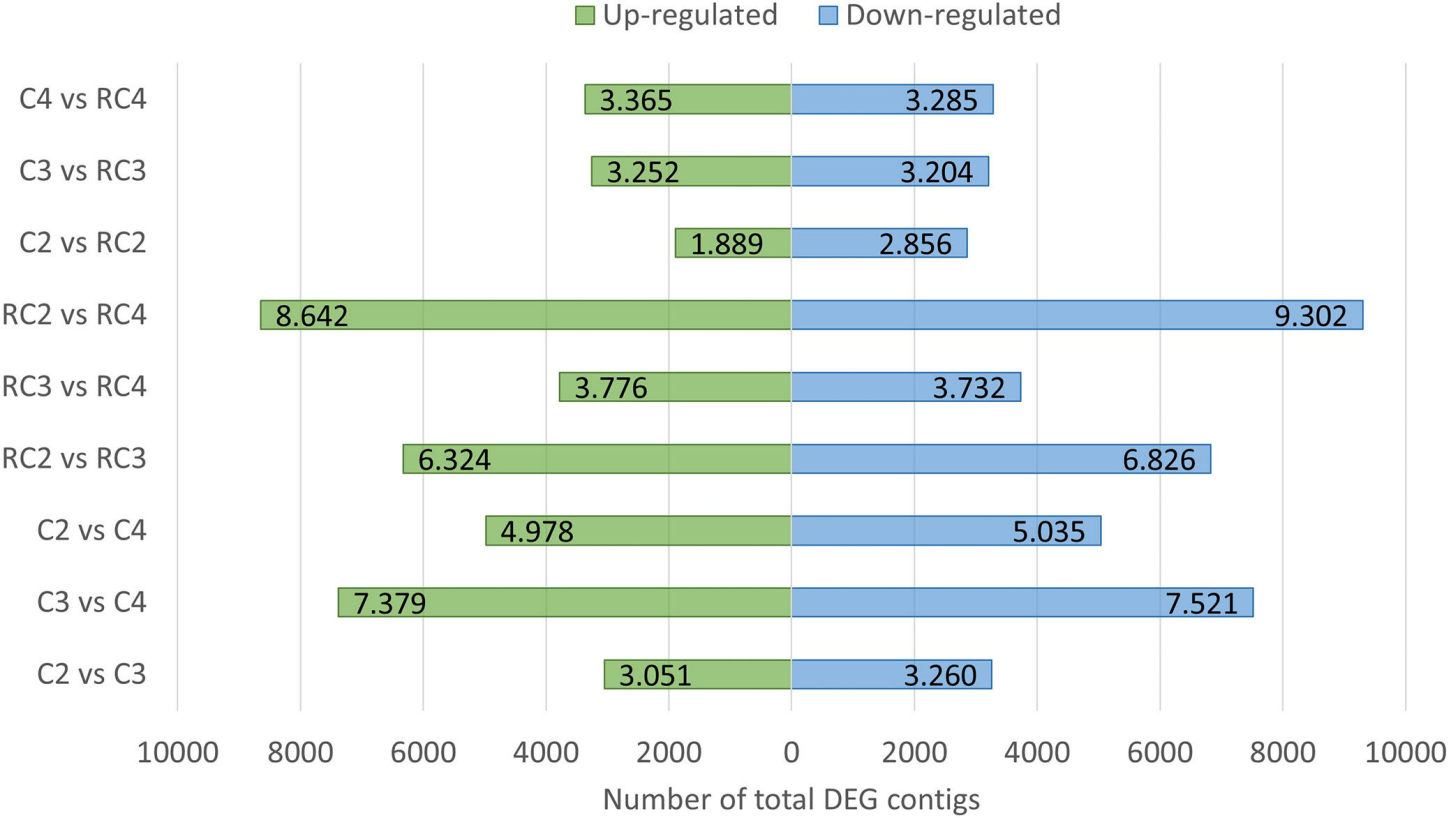
Up and down expressed genes in the different developmental and ripening stages. Contigs with a fold change ≥ [2] between the two samples are indicated. In each comparison, the first sample is the testing one, and the second is the control. The analysis was performed on 77,181 contigs, as 127,573 contigs were not considered for having at least one zero FPKM value in any of the libraries.

The number of differentially expressed contigs between pairs of fruit development ripening stages was analyzed by a fold change ≥2 (Figure 4). In most of these comparisons, the number of significant up and downregulated contigs was similar. However, the highest number of differentially expressed contigs (DEG) was observed when the developmental stages of C4 vs. C2, RC3 vs. RC2, and RC4 vs. RC2 were compared. On the other hand, the lowest number of differentially expressed contigs was observed between the receptacle, and a whole fruit sample from stage 2 was compared.

### Validation of RNAseq expression analysis

**Figure 5:**
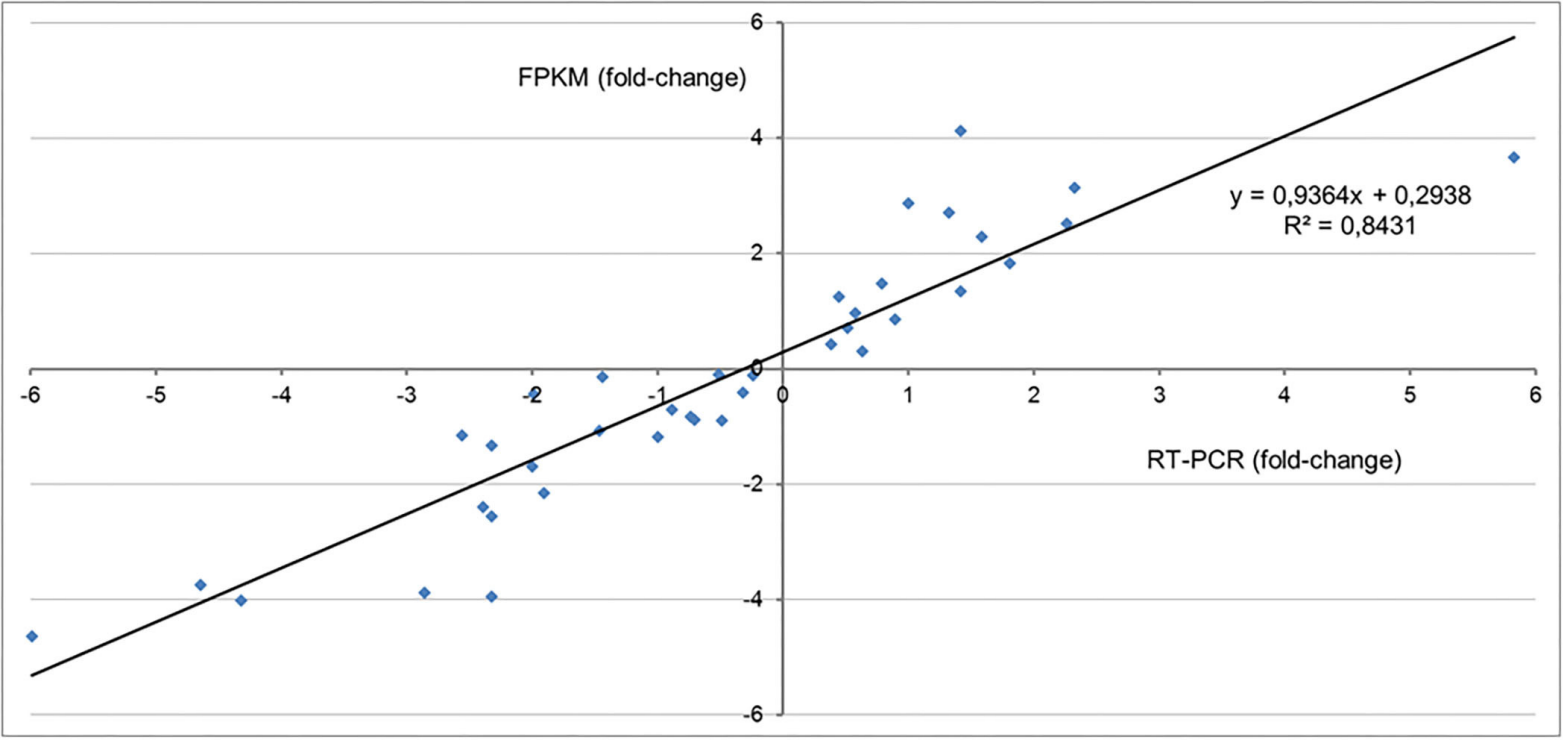
Validation of RNAseq analysis. The graph displays the relationship between the changes in expression determined by RT-qPCR analysis and FPKM data from the RNA seq analysis. A correlation value of 0.8 was determined. The ratio of changes in the transcriptional level of genes comparing two fruit ripening/developmental stages was obtained from the RNAseq data [log2(FPKM)] and compared to expression data quantified by RT-qPCR analysis or data previously reported for the specie (Salvatierra et al., 2010) (For more information see Supplementary Figures 3A,B, and Supplementary Table 3).

Two complementary strategies were used to validate the expression analysis data. First, the FPKM values of a random set of genes were compared with expression values determined by RT-qPCR in the same RNA samples employed for the RNA seq library (r^2^ = 0.90) (Supplementary Table 3). Secondly, FPKM values of a selected group of ripening-related genes were compared to expression data previously reported for the specie (Salvatierra et al., 2010) (r^2^ = 0.78) (Supplementary Figure 3). The correlation values between the changes in expression levels for different genes during the development and ripening stages of *F. chiloensis* using both strategies was r^2^ = 0.84 (Figure 5), indicating a good correlation between FPKM values and RT-qPCR data.

### Data mining of differentially expressed genes

Data mining of differentially expressed genes was performed to identify relevant molecular pathways during the development and ripening of *F. chiloensis* fruit. In addition, receptacle and whole fruit samples were analyzed. Several molecular and biological processes involved in fruit ripening were determined as differentially expressed, which include the phenylpropanoid pathway, synthesis of flavonoids and anthocyanins, enzymes involved in cell wall metabolism, biosynthesis of aroma, and phytohormones. Transcription factors were also detected as differentially expressed in specific expression dynamics during fruit development and ripening. An apparent molecular fine-tuning is observed in terms of different isoforms of the same gene, as some are differentially expressed in different developmental stages and tissues, but in general, functional redundancy is observed.

**Figure 6:**
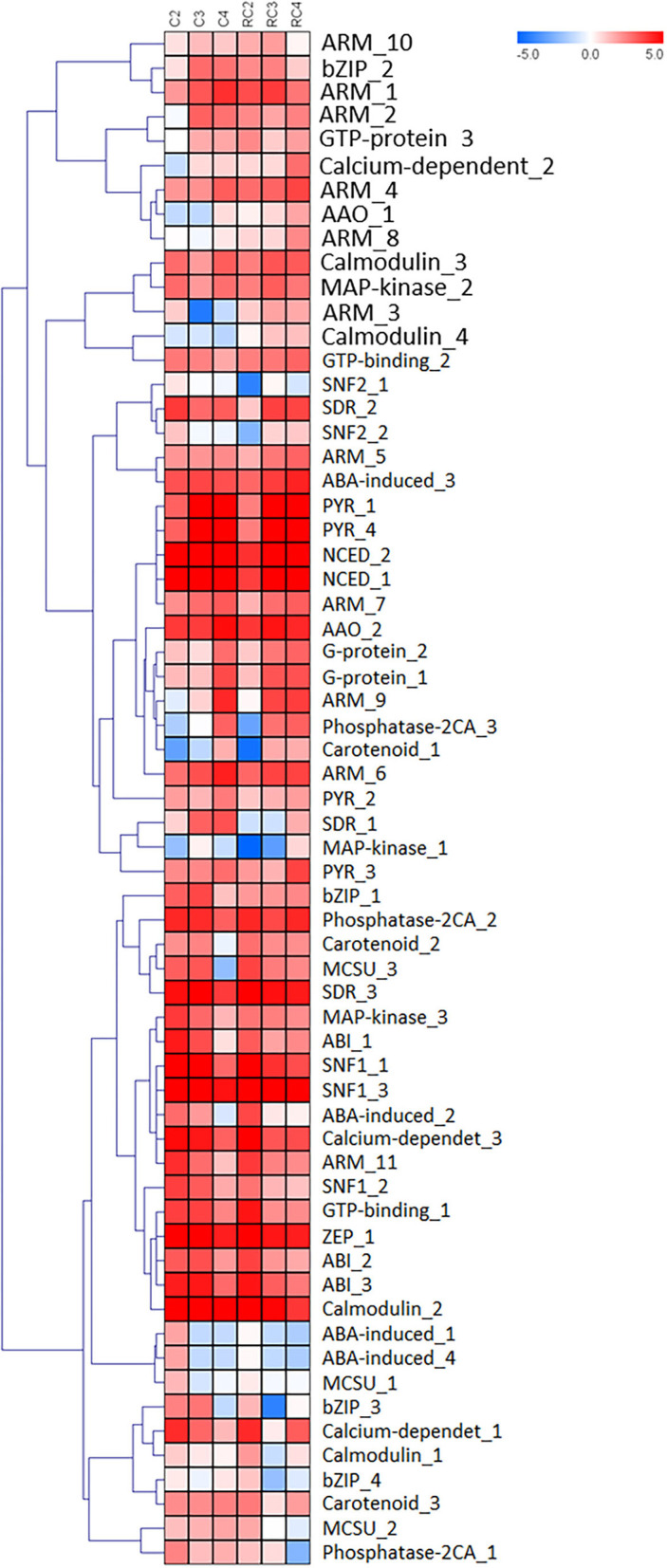
HeatMap for genes involved in the synthesis of Abscisic acid and receptors. Log2 is calculated to the FPKM value for each gene to delimit extreme values and used to scale expression. To perform hierarchical clustering for fruit developmental stage and tissue type (receptacle and receptacle without achene), we selected the Pearson Correlation distance metric and Average linkage clustering. Levels of decreased expression are shown in blue and increased expression is shown in red.

Based on the FPKM value, 39 tentative contigs corresponding to genes related to ABA regulation were analyzed by comparing fruit development stages. RNA was extracted from the whole fruit or receptacle without achenes (Figure 6). A differential expression pattern between early (C2) and late developmental stages (C3 and C4) was observed. Two main cluster groups can be observed in the heatmap. Genes involved in the synthesis of ABA can be observed in one group, and those genes involved in ABA receptor genes are mostly grouped in the other. Interestingly, three genes, calmodulin, MAP kinase, and phosphatase 2CA, present the same level of transcripts accumulated within all samples. The other 36 genes showed differential expression within the different tissues analyzed. Contigs for genes involved in ABA biosynthesis present high and differential accumulation of transcripts during the C2, C3, and C4 stages. Besides, contigs corresponding to ABA-receptors (PYR family and GCR2 family), phosphatases (PP2C), and protein kinases (SnRK2, CDPKs, and MAPKs), which interact in a regulatory network, are also differentially expressed. Interestingly, some genes are expressed in one type of tissue sample but not the other. For example, four genes (SNF2-1; ABA-induced 1; ABA-induced 4; MCSU1) are expressed only in the whole fruit, and four genes (ARM8; Calmodulin 4; MAPkinase 1; AAO-1) are only expressed in a receptacle. Additionally, 16 genes (ARM_5; ABA induced_3; PYR_1; PYR_4; NCED_2; NCED_1; ARM_7; AAO_2; ARM_6; PYR_3; SDR_3; Calcium dependent_3; SNF1_1; SNF1_3; ZEP_1; Calmodulin_2) show similar expression patterns, but 17 different genes (ARM_10; bZIP_2; ARM_1; ARM_2; GTP-protein_3; Calcium dependent_2; AAO_1; ARM_8; ARM_3; SNF2_2; Phosphatase 2CA_3; Carotenoid_1; SDR_1; Carotenoid_2; MCSU_3; MCSU_2; Phosphatase 2CA_1) are quantitatively expressed in the different developmental and ripening stages.

**Figure 7:**
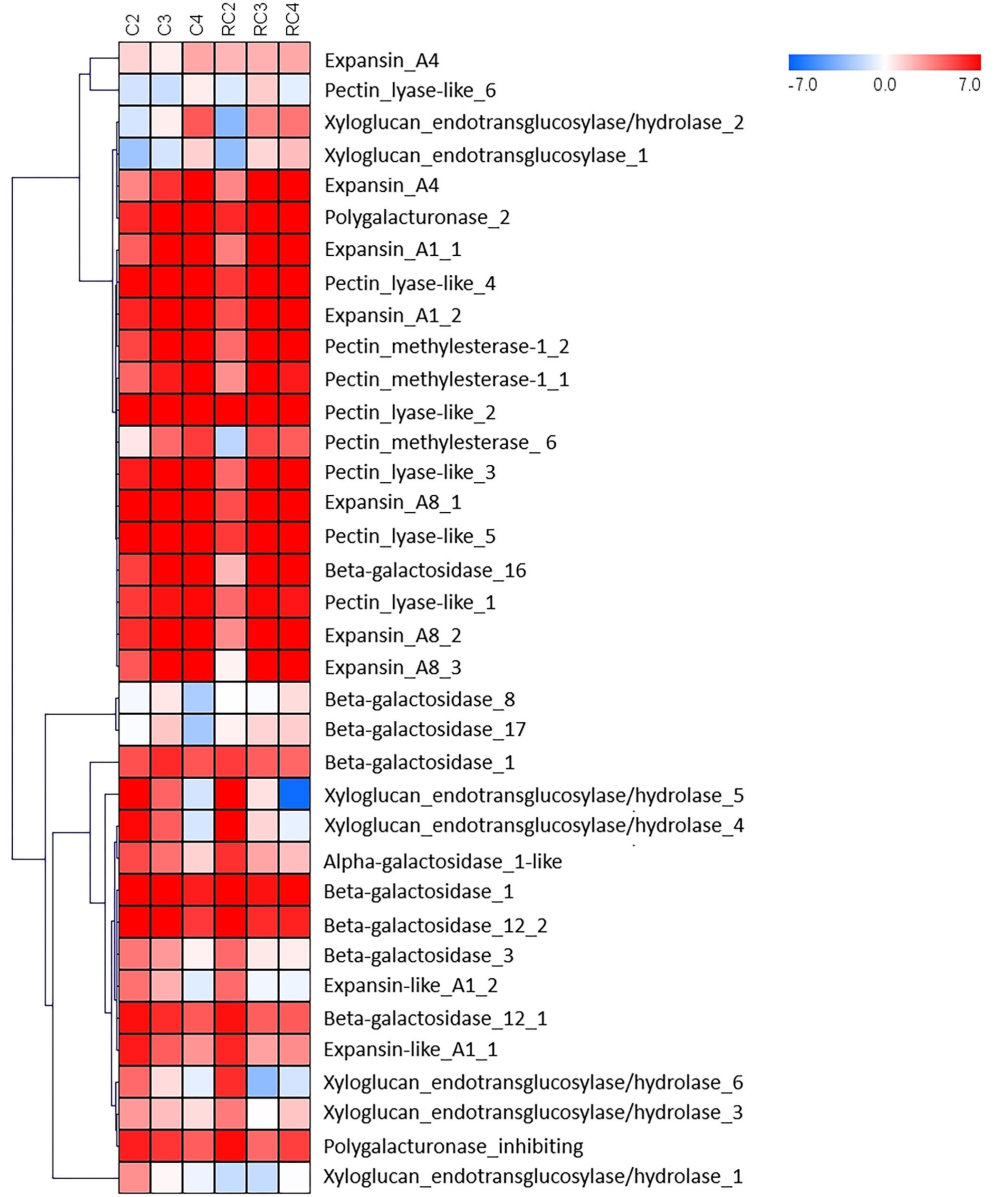
HeatMap for genes involved in the dynamics of the cell wall. Log2 is calculated to the FPKM value for each gene to delimit extreme values and used to scale expression. To perform hierarchical clustering for fruit developmental stage and tissue type (receptacle and receptacle without achene), we selected the Pearson Correlation distance metric and average linkage clustering. Levels of decreased expression are shown in blue and increased expression is shown in red.

When 36 DEG genes related to cell walls were analyzed (Figure 7), two cluster groups could be distinguished from the heatmap (Figure 7). Several members of the pectin lyase gene family are in one group, and members of the XTH gene family are in the other cluster group. Also, it can be observed that the two groups differed in the level of expression of the genes. One group is constituted by genes mostly over-expressed during fruit development and ripening, and the other group showed genes that are differentially expressed in one of the fruit stages. The expression pattern showed differences between early (C2) and late developmental stages (C3 and C4) but with divergence concerning fruit tissue. A higher expression level is observed for cell-wall-related genes in receptacles during the final stages of fruit development (RC3 and RC4).

Interestingly, 9 out of 36 genes showed no expression in all ripening stages. On the other hand, the EXP and P.L. grouped together showed similar expression patterns. However, they displayed a lower expression level than PME genes.

**Figure 8:**
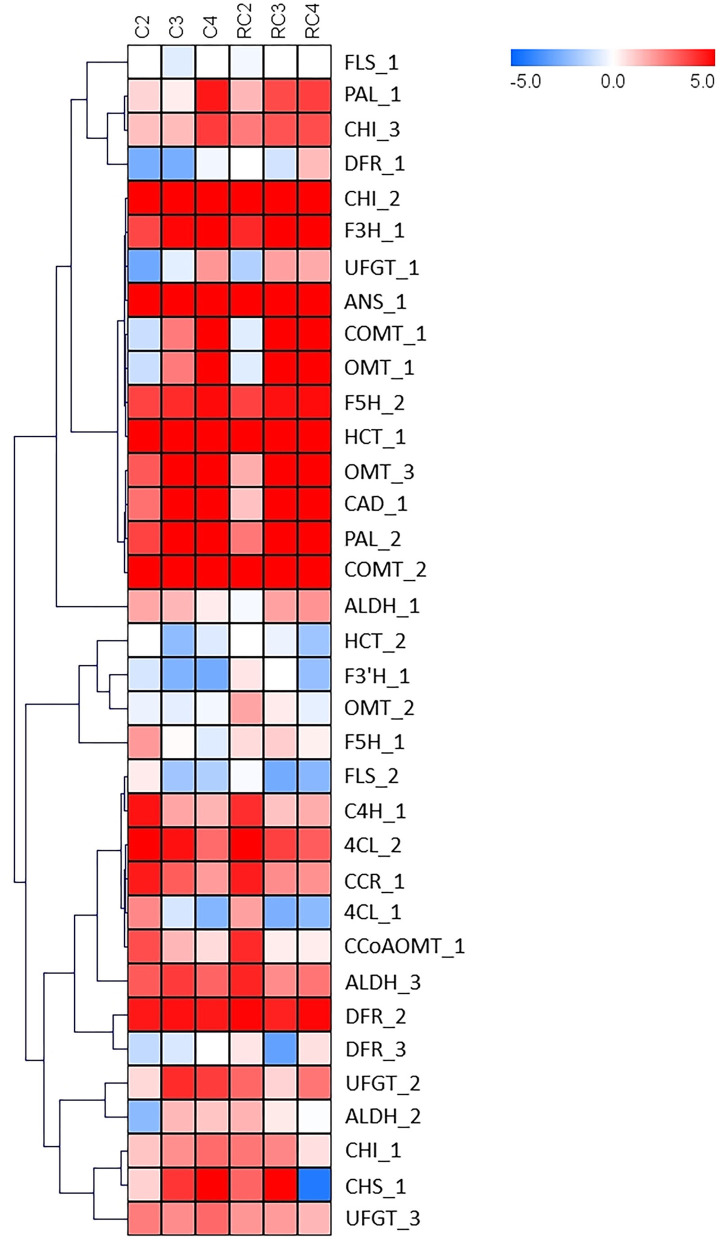
HeatMap for genes involved in the synthesis of flavonoids. Log2 is calculated to the FPKM value for each gene to delimit extreme values and used to scale expression. To perform hierarchical clustering for fruit developmental stage and tissue type (receptacle and receptacle without achene), we selected the Pearson Correlation distance metric and Average linkage clustering. Levels of decreased expression are shown in blue and increased expression is shown in red.

**Figure 9:**
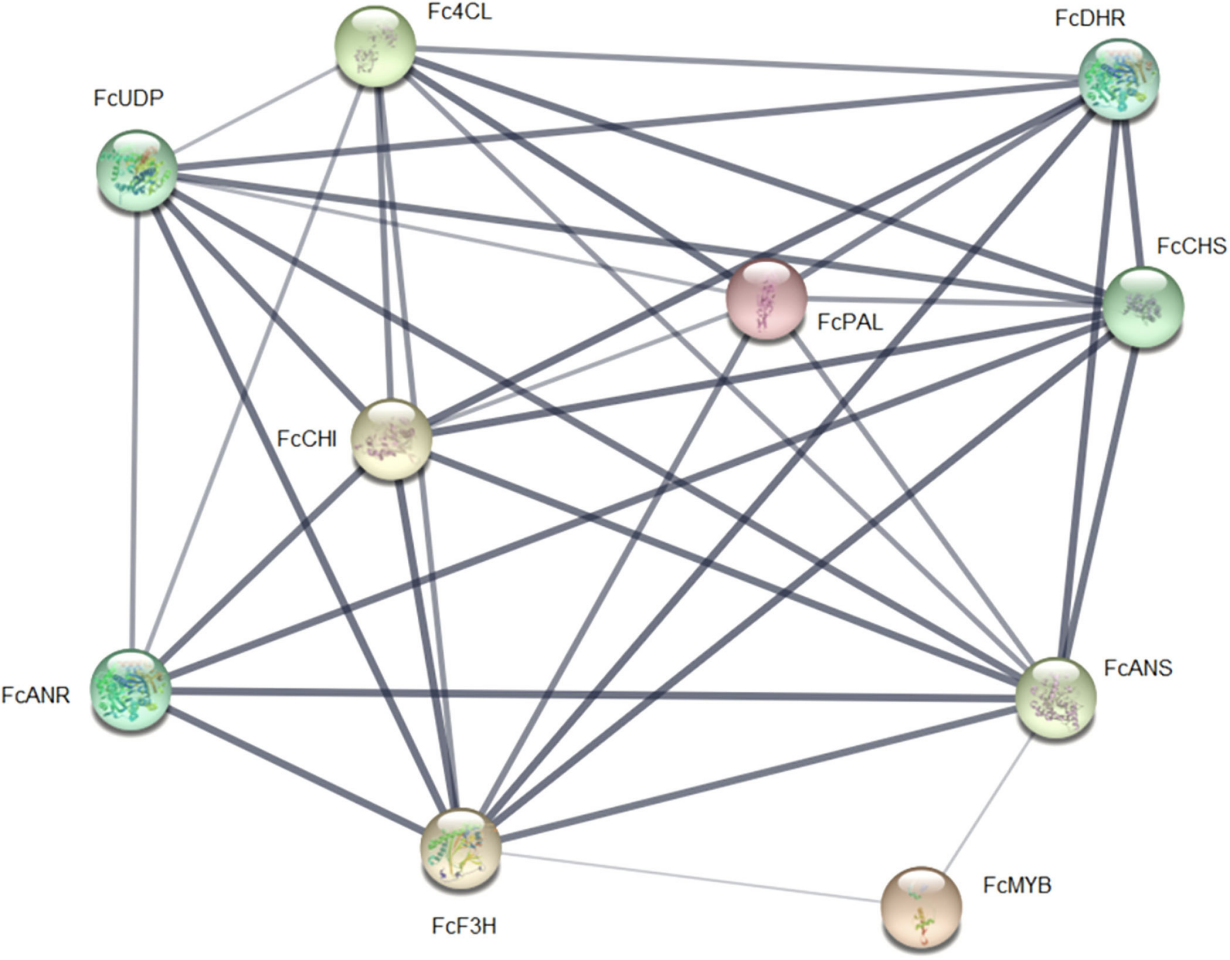
Network of gene interactions among genes differentially expressed from the phenylpropanoid and flavonoid pathways and FcMYB using STRING. Each node represents the interaction described in the literature between proteins from the phenylpropanoid and flavonoid pathways and FcMYB1, whose transcripts are differentially expressed in the RNAseq.

When genes involved in the phenylpropanoid and flavonoid pathways were analyzed, several genes showed a differential expression pattern (Figure 8). Key genes involved in the phenylpropanoid pathway are upregulated during the late stages of ripening (C3 and C4). The genes encoding for PAL isoforms, the first committed enzyme of this pathway, and COMT I, involved in lignin biosynthesis, demonstrated this expression profile. Besides, genes involved in the synthesis of flavonoids showed a remarkable accumulation of transcripts for genes associated with the biosynthesis of anthocyanins throughout ripening. Two main clusters can be observed in the heatmap, but one of them shows four sub-clusters. Interestingly, the genes involved in synthesizing flavonoids are on in one sub-cluster. When differentially expressed genes from receptacle were analyzed using STRING, there was an interaction between those genes (Figure 9). Interestingly, FcMYB was linked to FcANS and FcF3H.

## Discussion

As the mother of the commercial F. x ananassa, the Chilean strawberry has become an interesting species for gene discovery. Fruit development and ripening in strawberries is an intricate process at the molecular level (Moya-León et al., 2019). Several genes have been identified and characterized for our group in this Chilean native species as involved in the softening process, which include pectin methylesterase (Figueroa et al., 2008), Expansins (Gaete-Eastman et al., 2015), Xyloglucan endotransglycosylase/hydrolase (XTH) (Opazo et al., 2010, 2013; Méndez-Yañez et al., 2017), and Rhamnogalacturonan I lyases (Méndez-Yañez et al., 2020). Other genes/enzymes have been described in the commercial *F*. x *ananassa*, which presented a high index of similarity with its maternal species (Civello et al., 1999; Harrison et al., 2001; Benítez-Burraco et al., 2003; Castillejo et al., 2004; Rosli et al., 2004; Santiago-Doménech et al., 2008; Villarreal et al., 2008; García-Gago et al., 2009; Quesada et al., 2009; Molina-Hidalgo et al., 2013).

RNAseq has been used to unravel intricate processes at the molecular level, such as fruit development and ripening in commercial strawberries (Sanchez-Sevilla et al., 2017; Gu et al., 2019; Zhao et al., 2021). The description of a massive number of genes in the Chilean strawberry is described for the first time. The over-expression of metallothionein-like proteins was detected during the quality check of the NGS libraries. The same discovery has been reported in similar studies performed during the development and ripening of pineapple and other Fragaria fruit species (Nam et al., 1999; Moyle et al., 2005).

Gene ontology analyses provide a broad view of the transcriptional changes occurring during a particular biological event. In the case of *F. chiloensis*, most of the transcripts have been included within categories previously defined to be crucial for the development of fruit ripening. The “catalytic activity” and “binding” subcategories are strongly represented in the category of Molecular Function, which represents the active participation of multiple enzymes during fruit development and ripening, as well as the selective interaction between molecules and their specific ligands. In the same category, two other subcategories with high hits were “transporter” and nucleic acid binding transcription factor activities that predict an active transcriptional activity during fruit development and dynamic transport of molecules between compartments. In the category of cellular components, some subcategories were highly represented, including “organelle” and “membrane,” which again represent the active participation of several organelles during the strong metabolic activity normally observed during fruit ripening. Similarly, the same categories were found when a suppression subtractive hybridization library was built (Pimentel et al., 2010).

In the case of the biological process category, the most represented subcategory is the metabolic process, and other important subcategories are the response to stimulus, biological regulation, cellular process, and developmental process. As a whole, this classification of the *F. chiloensis* transcripts perfectly represents the active metabolic activity developed during fruit ripening. This developmental process involves many cell compartments and the active movement of intermediates within organelles. The comparisons in the cluster analysis for the different sample stages showed the highest number of differentially expressed contigs (DEG) for the pair's conditions RC3 vs. RC2; RC4 vs. RC2; and C4 vs. C2, which could indicate that major changes in transcription are taking place between the C2 stage and the C3/C4 stages.

The massive number of transcripts obtained in RNAseq provides the opportunity to find other gene family members. In this study, several members of gene families related to cell walls were identified in the analysis of the RNAseq. Thus, members of the P.L., XTH, PME, β-Gal, and Exp family genes showed differences in the accumulation of transcripts during fruit ripening. A possible explanation of these results could be that many of these enzymes participate both in the biosynthesis and degradation of the cell wall, so some isoforms of genes associated with hemicellulose and pectin are early expressed. In contrast, other isoforms are lately expressed, modifying similar cell wall components but most probably breaking down glycosidic bonds. For instance, six new XTH isoforms were identified as differentially expressed during fruit development and ripening in *F. chiloensis*; one XTH showed high similarity to the gene previously reported by Opazo et al. (2010). Several members of gene families are differentially expressed simultaneously during fruit development and ripening. In this sense, a genome-wide analysis in the diploid strawberry *F. vesca* indicated 26 different XTHs, reported as differentially expressed in different tissues. However, only four displayed an accumulation of transcripts during fruit ripening (Opazo et al., 2017).

Interestingly, XTH presented two different types of activities. Different enzyme isoforms could reflect the need for hydrolysis or the modification in the extension of the xylan structure in the cell wall. Additionally, five new P.L.s, one P.G., and five new expansions were found as differentially expressed, but three new PME and eight β-Gal were also reported. There is no doubt that during fruit development and ripening, several events are influenced by the dynamics of the cell wall, which requires the participation of several different enzymes.

The plant hormones ABA and auxins play a key role during strawberry fruit development and ripening in *F. chiloensis* (Moya-León et al., 2019). Nicely, most of the genes involved in synthesizing both plant hormones are present in the RNAseq library and are differentially expressed. The results showed contigs corresponding to ABA-receptors (PYR family and GCR2 family), phosphatases (PP2C), and protein kinases (SnRK2, CDPKs, and MAPKs), which interact in a regulatory network where several components are differentially expressed. The genes SDR, MCSU, and AAO, which participate in the biosynthesis of ABA, were also found in the RNAseq library. The role of these plant hormones has been studied in commercial strawberries. A microarray chip was used, and several cell wall-related genes presented a high fold change after exogenous application of auxins and ABA (Medina-Puche et al., 2016). The study of the role of different plant hormones was analyzed in *F*. x *ananassa* cv. Seolhyang, and apart from the role of ABA in fruit ripening, it was proposed that *FaMYB10* is key in regulating strawberry ripening (Kim et al., 2019). In the late stage of fruit ripening, ABA plays a role, and in *F. chiloensis, it* also plays a role in softening (Mattus-Araya et al., 2022a) and color (Mattus-Araya et al., 2022b).

Another interesting molecular pathway for this species is the biosynthesis of flavonoids. Flavonoids are associated with the red fruit color, mainly pelargonidin-3-glucoside, but *F. chiloensis* is a white-pinkish fruit, even though it accumulates anthocyanins as cyanidin-3-glucoside (Simirgiotis et al., 2009). Interestingly, most of the genes presented similar expression patterns when the whole fruit was compared to the receptacle sample, but interestingly, a sub-cluster group containing all the genes for synthesizing flavonoids was all “on” at the late time of ripening (RC3 and RC4). Three different transcription factors have been reported to be involved in regulating fruit color (Shaart et al., 2012). *FcMYB* plays a role in the appearance of the red color in the Chilean strawberry during the orthologs in the commercial *F*. x *ananassa* cv. Camarosa (*FaMYB1*) is downregulated (Salvatierra et al., 2013). The transcription factors MYB5 and MYB11 were also identified in the RNAseq and MYB1. The interference in the expression of *FcMYB1* showed the downregulation of LAR and ANR and the upregulation of ANS and UFGT, which allowed the synthesis of proanthocyanidins (Salvatierra et al., 2013). String showed the close role of MYB with genes from the flavonoid molecular pathways during fruit ripening. Even though the two other members of this regulation cluster (bHLH and WD40) were also identified, in most of the samples, their FPKM values were below the cutoff.

## Conclusion

The RNAseq library analyzed the massive transcriptional changes during the development and ripening of F. chiloensis fruit. The strategy generated a comprehensive database of expressed sequences during the process for the white Chilean strawberry fruit. Data mining of annotated sequences to G.O. terms classified into “cell wall,” “phenylpropanoid-flavonoid,” and “ABA-regulation” revealed a biologically meaningful reprogramming at different fruit tissues and ripening stages. Several members of different gene families were identified as differentially expressed, which may play a role in the cell wall's dynamics and fruit color development.

## Data availability statement

The datasets presented in this study can be found in online repositories. The names of the repository/repositories and accession number(s) can be found in the article/Supplementary material.

## Author contributions

The present work was conceived and designed by RH, CG-E, and MM-L. SM prepared the RNA samples for sequencing. CG-E, YS, DU, SM, and MM-L contributed to collecting information. YS, CG-E, MM-L, and RH participated in the design of figures. YS prepared most of the tables and figures. MM-L and RH participated actively in the writing and discussion of the manuscript. All authors participated sufficiently in the work to take public responsibility for appropriate portions of the content, read, edited, and approved the final manuscript.

## Funding

Thanks to ANID-FONDECYT Regular grants 1210948 (MM-L) and 1201011 (RH), and ANID-ACT210025 for financial support. The funders had no part in the design of the study or collection, analysis, interpretation of data, and in writing the manuscript.

## Conflict of interest

The authors declare that the research was conducted in the absence of any commercial or financial relationships that could be construed as a potential conflict of interest.

## Publisher's note

All claims expressed in this article are solely those of the authors and do not necessarily represent those of their affiliated organizations, or those of the publisher, the editors and the reviewers. Any product that may be evaluated in this article, or claim that may be made by its manufacturer, is not guaranteed or endorsed by the publisher.
