# Supplementary material for: RNAseq, transcriptome analysis and identification of DEGs involved in development and ripening of Fragaria chiloensis fruit
Source: Front Plant Sci. 2022 Sep 20;13:976901. doi: 10.3389/fpls.2022.976901 (PMC9530326; doi:10.3389/fpls.2022.976901)
Supplement: Supplementary file 1 [file Image_1.pdf]

Supplementary Material

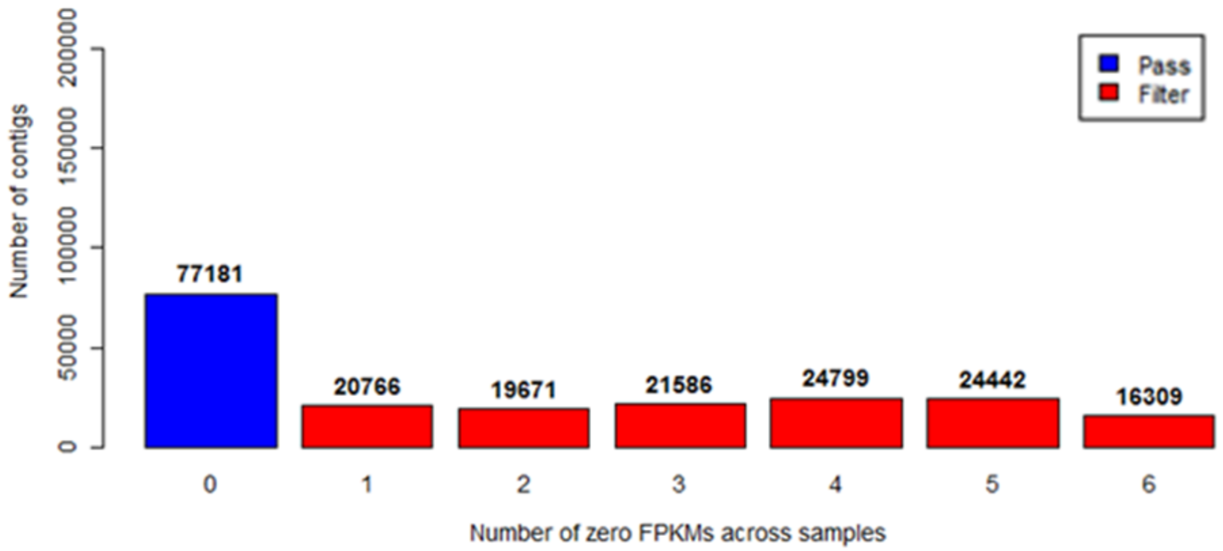

Supplementary Figure 1. Analysis of expression values of the contigs assembled from *F. chiloensis* libraries. The FPKM values of 127,573 contigs were compared within the different RNA-seq libraries. The contigs were classified according to the number of zero FPKM values in the samples. A total of 77,181 contigs continued in the analysis.
