# Supplementary figures and images for: RNAseq, transcriptome analysis and identification of DEGs involved in development and ripening of Fragaria chiloensis fruit
Source: Front Plant Sci. 2022 Sep 20;13:976901. doi: 10.3389/fpls.2022.976901 (PMC9530326; doi:10.3389/fpls.2022.976901)

# Supplementary Material

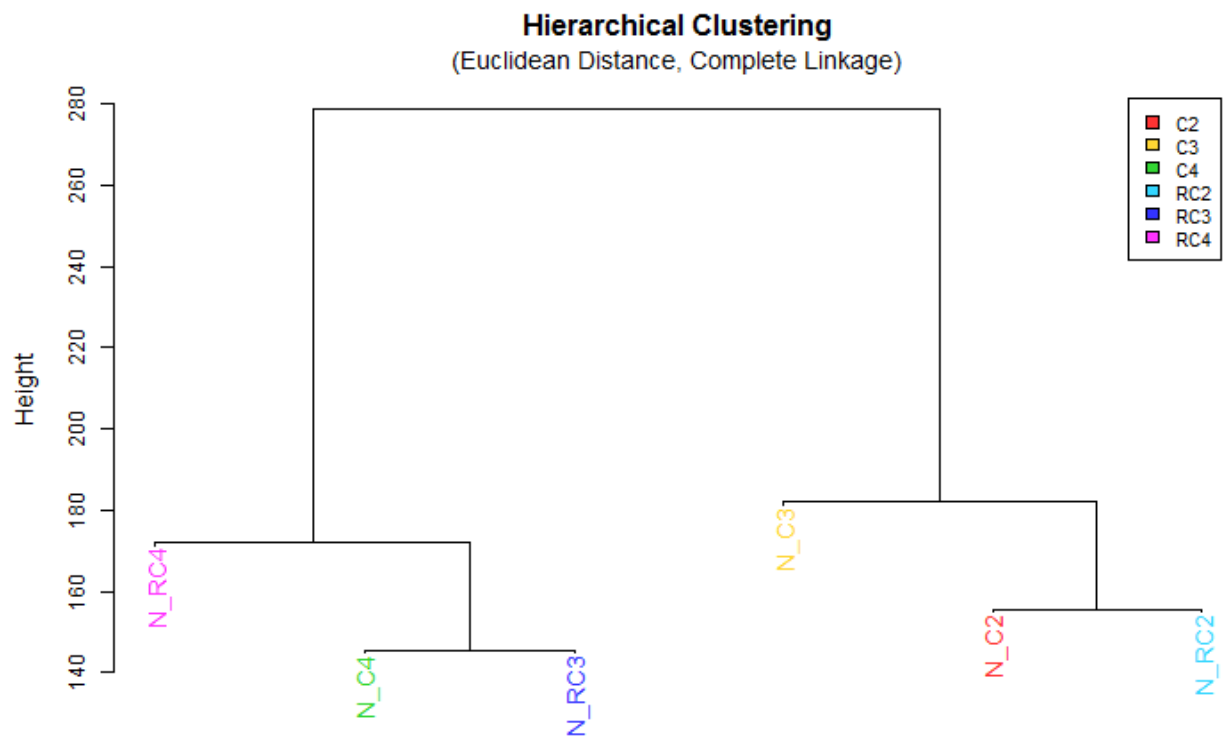

**Supplementary Figure 2.** Hierarchical clustering of RNA-seq libraries.

Supplement: Supplementary file 2 [file Image_2.pdf]
