# Supplementary material for: RNAseq, transcriptome analysis and identification of DEGs involved in development and ripening of Fragaria chiloensis fruit
Source: Front Plant Sci. 2022 Sep 20;13:976901. doi: 10.3389/fpls.2022.976901 (PMC9530326; doi:10.3389/fpls.2022.976901)
Supplement: Supplementary file 3 [file Image_3.pdf]

Supplementary Material

A

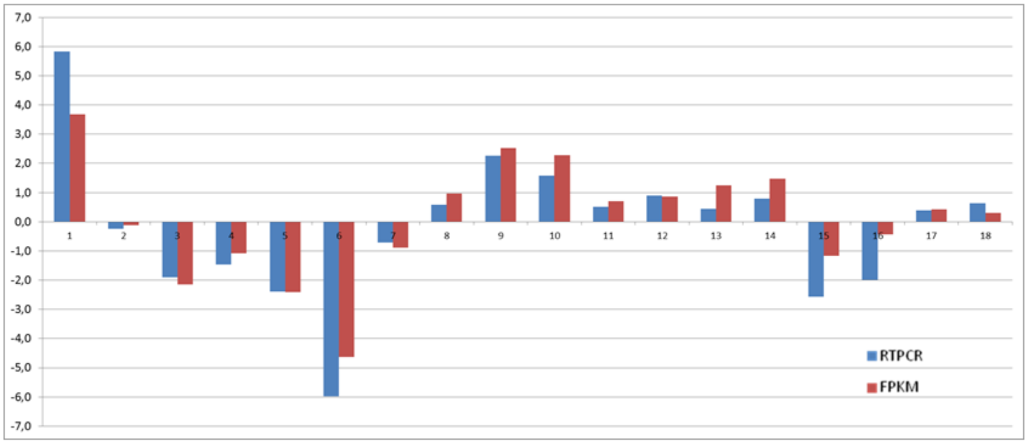

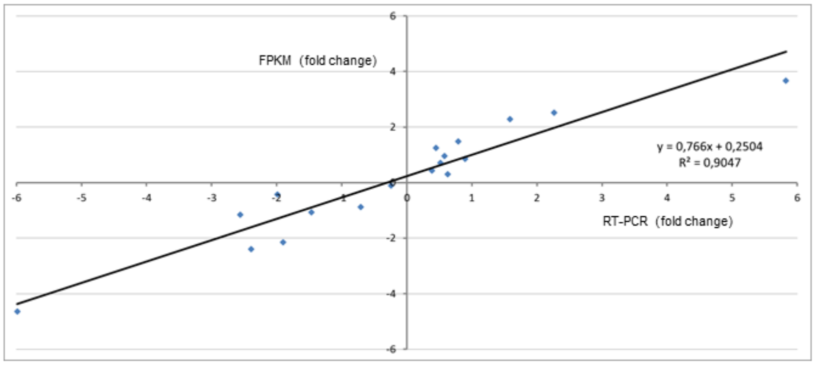

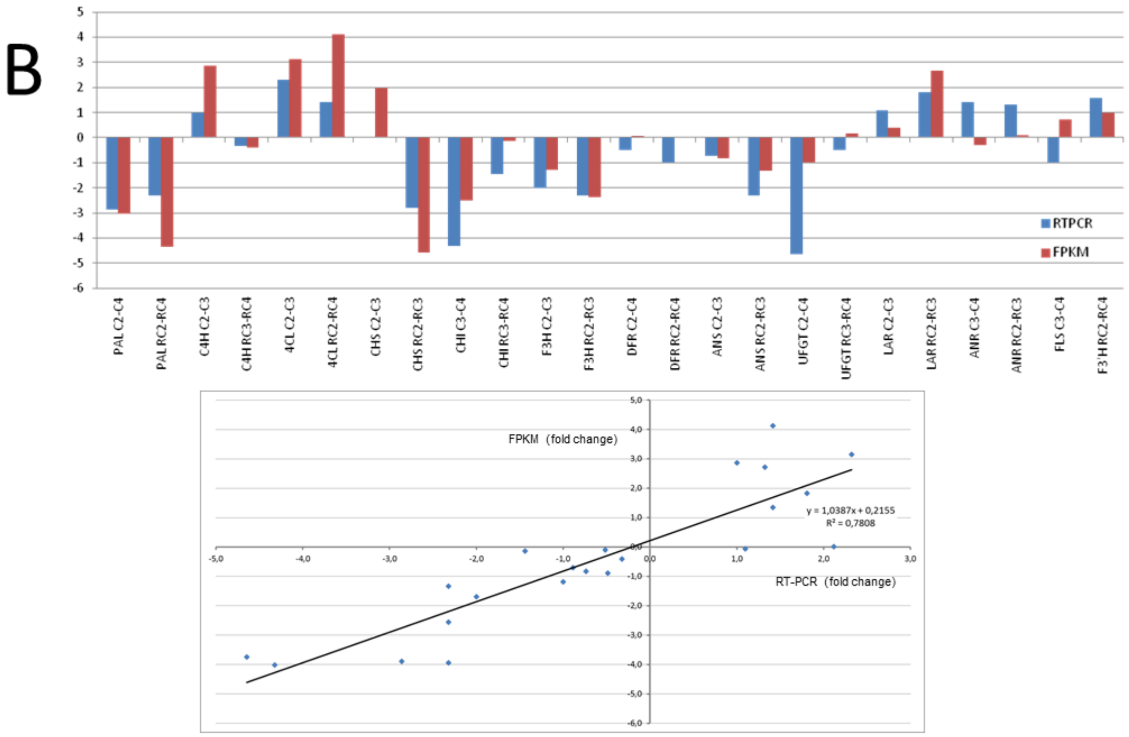

Supplementary Figure 3. Validation of RNAseq analysis. Each graph displays the relation between the changes in expression determined by RT-qPCR analysis and FPKM data from the RNA seq analysis. The ratio of changes in the transcriptional level of genes comparing two fruit ripening/developmental stages were determined from the RNAseq data [log2(FPKM)] and compared to expression changes quantified by RT-qPCR analysis (A), or data previously reported in the literature for the species (B). Correlation graphs describing the transcriptional changes obtained from log2(FPKM) measurements and qPCR analysis were incorporated under each graph. Genes analyzed in (A) correspond to: 1, *asABI5* (C2-C3); 2, *asABI5* (RC3-RC4); 3, *asANS* (C2-C4); 4, *asANS* (RC2-RC4); 5, *asPAL* (C2-C3); 6, *asPAL* (RC2-RC3); 7, *asPP2C* (C2-C3); 8, *asPP2C* (C3-C4); 9, *CCR1* (C2-C4); 10, *CCR1* (RC2-RC4); 11, *UFGT* (C2-C3); 12, *UFGT* (RC2-RC4); 13, *NCED* (C2-C3); 14, *NCED* (RC2-RC3); 15, *PYL4* (C3-C4); 16, *PYL4* (RC3-RC4); 17, *SNRK2* (C3-C4); 18, *SNRK2* (RC3-RC4). Correlation analysis indicate $r^2 = 0.90$. Genes analyzed in (B) correspond to *PAL, C4H, 4CL, CHS, CHI, F3H, DFR, ANS, UFGT, LAR, ANR, FLS, F3´H* and qPCR data was obtained from Salvatierra et al. (2010) ($r^2 = 0.78$). C samples correspond to *F. chiloensis* complete fruit samples, meanwhile RC samples correspond to receptacle samples.
