# Supplementary material for: RNAseq, transcriptome analysis and identification of DEGs involved in development and ripening of Fragaria chiloensis fruit
Source: Front Plant Sci. 2022 Sep 20;13:976901. doi: 10.3389/fpls.2022.976901 (PMC9530326; doi:10.3389/fpls.2022.976901)
Supplement: Supplementary file 4 [file Table_1.pdf]

Supplementary Material

Supplementary Table 1. *Fragaria chiloensis* transcriptome: sequencing outputs per library.

Library sample	Total read pairs	Read length	Total read bases
C2	70,813,054	101 bp	7,150,118,454
C3	94,976,154	101 bp	9,592,591,554
C4	60,665,022	101 bp	6,127,167,222
RC2	62,787,196	101 bp	6,341,506,796
RC3	69,560,234	101 bp	7,025,583,634
RC4	76,532,918	101 bp	7,729,824,718

RNA samples were obtained from *Fragaria chiloensis* fruit at different development and ripening stages. RNA samples were obtained from whole fruit (C2, C3, C4) and from fruit without achenes (RC2, RC3, RC4). Stage 2 corresponds to large green fruit, stage 3 to turning fruit, and stage 4 to ripe fruit. Illumina HiSeq2000 sequencing was applied for *de novo* transcriptome analysis. The information of raw data per each library is described.
