# Supplementary material for: RNAseq, transcriptome analysis and identification of DEGs involved in development and ripening of Fragaria chiloensis fruit
Source: Front Plant Sci. 2022 Sep 20;13:976901. doi: 10.3389/fpls.2022.976901 (PMC9530326; doi:10.3389/fpls.2022.976901)
Supplement: Supplementary file 5 [file Table_2.pdf]

Supplementary Material

Supplementary Table 2. Comparison of assembled sequences and those previously reported for *Fragaria chiloensis* in GenBank

Sequence	GenBank	RNAseq transcript id	% id	Alignment length	E-value
Fc_AAT1	FJ548610.1	comp6_c0_seq1	99.85	1353	0.0
Fc_MADSbox1	GQ398009.1	comp135_c0_seq2	99.81	1060	0.0
Fc_MYB1	GQ867222.1	comp1249_c0_seq1	99.65	564	0.0
Fc_PG1	EF441274.1	comp47_c0_seq1	99.84	1218	0.0
Fc_PL1	EF441273.1	comp34_c0_seq2	99.10	1217	0.0
Fc_XTH1	GQ280283.1	comp279_c0_seq2	98.98	886	0.0
Fc_XTH2	GQ280284.1	comp654_c0_seq1	99.56	919	0.0
