# Supplementary material for: RNAseq, transcriptome analysis and identification of DEGs involved in development and ripening of Fragaria chiloensis fruit
Source: Front Plant Sci. 2022 Sep 20;13:976901. doi: 10.3389/fpls.2022.976901 (PMC9530326; doi:10.3389/fpls.2022.976901)
Supplement: Supplementary file 6 [file Table_3.pdf]

Supplementary Material

Supplementary Table 3. Primers employed for RT-qPCR analysis performed for validation of expression data.

Sequence	RNAseq transcript id		Primer sequence (5' →3')
asABI5	comp16733_c0_seq1	Fw	GCCTCCAGGTTCGTCAGAAG
		Rev	AAGCAGATCACCCTCAGTGC
asPAL	comp262_c0_seq2	Fw	TGCATTCCTATAGAGGGTTCTTGA
		Rev	GGCACAAATGTGTCTTTACCCA
asPP2C	comp7688_c0_seq2	Fw	TGCGACAGGTACCATTGTCC
		Rev	CAAGGTCGTTCGGGGAAGAA
CCR1	comp9194_c0_seq1	Fw	TGGTAGGTGAGTGGTAGGCT
		Rev	CTGTGATCAGCAGGCATCTTTG
UFGT	comp2402_c0_seq10	Fw	ACCTAGGGTAGCAGCGTACA
		Rev	GTCATGCTCTTGCTGGCAAC
NCED	comp35890_c0_seq2	Fw	TCCTCGACTGTTCAGCCTTTC
		Rev	TGGGAGTGAGGGTACATCCG
PYL4	comp7632_c0_seq1	Fw	CTTGTGGTGGTGTTCTGGGT
		Rev	CTCCAACGGCAAACACAGAC
SNRK2	comp3518_c0_seq1	Fw	GTTTGGAATTGGATCGGGCG
		Rev	GGTCTGCTTGTCGGTCATCA
